# Nutritional status, intestinal parasite infection and allergy among school children in Northwest Ethiopia

**DOI:** 10.1186/1471-2431-13-7

**Published:** 2013-01-12

**Authors:** Bemnet Amare, Jemal Ali, Beyene Moges, Gizachew Yismaw, Yeshambel Belyhun, Simon Gebretsadik, Desalegn Woldeyohannes, Ketema Tafess, Ebba Abate, Mengistu Endris, Desalegn Tegabu, Andargachew Mulu, Fusao Ota, Bereket Fantahun, Afework Kassu

**Affiliations:** 1 Collage of Medicine and Health Sciences, Department of Medical Biochemistry, University of Gondar, Gondar, Ethiopia; 2 Collage of Medicine and Health Sciences, Department of Microbiology Immunology and Parasitology, University of Gondar, Gondar, Ethiopia; 3 Collage of Medicine and Health Sciences, Department of Epidemiology and Biostatistics, University of Gondar, Gondar, Ethiopia; 4 Seto Medical Check Clinic, Takamatsu, Japan

**Keywords:** Nutritional status, Parasite infection, Allergy, Ethiopia

## Abstract

**Background:**

Parasitic infections have been shown to have deleterious effects on host nutritional status. In addition, although helmintic infection can modulate the host inflammatory response directed against the parasite, a causal association between helminths and allergy remains uncertain. The present study was therefore designed to evaluate the relationship between nutritional status, parasite infection and prevalence of allergy among school children.

**Methods:**

A cross sectional study was performed involving school children in two elementary schools in Gondar, Ethiopia. Nutritional status of these children was determined using anthropometric parameters (weight-for-age, height-for-age and BMI-for-age). Epi-Info software was used to calculate z-scores. Stool samples were examined using standard parasitological procedures. The serum IgE levels were quantified by total IgE ELISA kit following the manufacturer’s instruction.

**Result:**

A total of 405 children (with mean age of 12.09.1 ± 2.54 years) completed a self-administered allergy questionnaire and provided stool samples for analysis. Overall prevalence of underweight, stunting and thinness/wasting was 15.1%, 25.2%, 8.9%, respectively. Of the total, 22.7% were found to be positive for intestinal parasites. The most prevalent intestinal parasite detected was *Ascaris lumbricoides* (31/405, 7.6%). There was no statistically significant association between prevalence of malnutrition and the prevalence of parasitic infections. Median total serum IgE level was 344 IU/ml (IQR 117–2076, n = 80) and 610 IU/ml (143–1833, n = 20), respectively, in children without and with intestinal parasite infection (Z = −0.198, P > 0.8). The prevalence of self reported allergy among the subset was 8%. IgE concentration was not associated either with the presence of parasitic infection or history of allergy.

**Conclusion:**

The prevalence of malnutrition, intestinal parasitism and allergy was not negligible in this population. In addition, there was no significant association between the prevalence of allergy and their nutritional status, and parasite infection. Further research prospective observational and intervention studies are required to address the question of causality between nutritional factors, parasites, and allergy.

## Background

According to the World Health Organization (WHO), approximately 2 billion people are affected by helminthic infection worldwide [1]. These infections are responsible for high levels of morbidity and mortality, including iron-deficiency anemia, seizures, portal hypertension, and chronic diarrhea [1-3]. The prevalence of intestinal parasites is determined by the socioeconomic and health conditions, education and beliefs related to traditional health practices, as well as the presence of domestic animals in the home and contamination of water and food. Age is also an associated factor related to the individual’s immunologic status and behavioral patterns contributing to the high prevalence of intestinal parasites in children than in adults [4,5]. Children also are vulnerable to serious complications of helminthic infection, such as malnutrition, anemia, bowel obstruction, and learning disabilities [1,6,7].

Nutritional status is a key indicator of health assessment [8]. Malnutrition is a common health problem of African schoolchildren due to intestinal parasitic infections and many other factors [9].

The relation between the low prevalence of allergic disorders in poor subsistence societies compared to urbanized societies is not well understood. One explanation behind this is the high prevalence of intestinal parasitic infections, mainly helminthes, in rural setups of developing countries. Such infections have been postulated to prevent IgE mediated allergic diseases by blocking effector cell IgE receptors with parasite induced specific and polyclonal IgE or by production of the anti-inflammatory cytokine interleukin-10 [10]. Despite having been evaluated for decades, the role of parasite infection on allergy prevalence remains unclear [11]. Results range from no association [12] and negative association [13] to positive association [14] between parasite infection and allergy.

Undernutrition and parasitic diseases have a similar geographical distribution with the same people experiencing both insults together for much of their lives [6]. Parasitic infections are thought to contribute to child undernutrition through subtle reduction in digestion and absorption, chronic inflammation and loss of nutrients. In turn, undernutrition can make a person more susceptible to parasitic diseases, which causes a vicious cycle [1,6-9]. However, No comprehensive study has been conducted to elucidate nutritional status, prevalence of intestinal parasites and allergy, and the corresponding immunological correlates among school children in Gondar area of Ethiopia. This project, therefore, was conducted to generate useful information of public health importance by addressing the relationship between nutrition, parasitoses, and allergic disorders.

## Methods

### Study design and setting

This cross sectional study was conducted by involving 405 school children in two randomly selected governmental elementary schools in Gondar town, Ethiopia. Gondar town is located in the Northwestern part of Ethiopia with population of 200,000 [15]. The data collection was done over 10 consecutive days in January–February 2008.

### Sample size and sampling procedure

A single population proportion formula, [n = (Z α/2)^2^ p (1-p)], was used to estimate the sample size. The following assumptions were made: since there was no study conducted regarding this topic in the area, proportion was taken as 50% (p = 0.5), 95% confidence interval, margin of error 5% (d = 0.05). Computing with the above formula and 10% of contingency gives a total sample size of 422. Seventeen data of school children were incomplete and therefore excluded, leaving 405 (96%) for analysis.

All government elementary schools in a town were registered first and from the list two schools were selected as a study schools by simple random sampling methods. Stratified sampling method was used to select study participants. Stratification was made based on the grade the students were attending and then by sex to balance the male to female ratio. The study units were then selected using systematic sampling from each stratum. Written consent was obtained from their parents and the school principal to participate in the study. The study protocol was approved by the Research Ethics Committee of the University of Gondar.

### Data collection

A pre-tested and structured questionnaire in English and translated to Amharic [National language] was used to collect data. The questionnaire had both closed and open-ended questions. It was composed of three parts. The first part, which was the preamble, contained the objectives and significance of the study. The second part contained information on the socio-demographic characteristics of the study participants. The third part assessed self reported history of allergy. Participants were asked if they had been diagnosed by physician for asthma, hay fever, eczema or allergy of the skin, or other allergic conditions. School children were defined as having allergy if they answered “yes” to any of these questions and not having allergy if they answered “no” to all of these questions.

### Nutritional assessment

Body weight was determined to the nearest 0.1 kg on an electronic digital scale and height was measured to the nearest 0.1 cm. Body Mass Index (BMI), defined as the weight in kilogram of the individual divided by the square of the height in meter, was used to determine the nutritional status of the school children into severe malnutrition (BMI < 15.9 kg/m^2^), moderate malnutrition (BMI = 16–16.9 kg/m^2^), mild malnutrition (BMI = 17–18.4 kg/m^2^) and normal (BMI = 18.5-25 kg/m^2^) as recommended by WHO. Weight, height and BMI were also used to determine Z scores for weight-for-age, height-for-age and BMI-for-age using the NCHS/WHO reference data [16].

### Collection of stool specimens and examination for helminths

Student was advised to bring about 2gm of fresh stool and was given a labeled, clean, dry and leak proof stool cup. The samples were processed following standard procedures in clean leak-proof stool cups. Just after collection they were examined by two senior clinical laboratory technicians independently for intestinal parasites following direct and concentration methods [17]. The Kato method was also used for estimation of number of eggs per gram of stool to determine intensity of infection burden at an individual level. Students found positive for intestinal parasites were given appropriate anti-parasite chemotherapy by a medical doctor.

### Serum IgE determination

The serum IgE levels were quantified by total IgE ELISA kit (IBL Immunobiological Laboratories, Hamburg, Germany) following the manufacturer’s instructions. In brief, 10 ml serum samples or standard IgE were pipetted in duplicates into wells of micro-titer plates pre-coated with monoclonal mouse antihuman IgE antibody together with peroxidase conjugated antihuman IgE. After incubation for 30 minute at room temperature the plates were rinsed with diluted wash buffer to remove unbound material. Then a substrate solution (tetra-methylbenzidine) was pipette and incubated for 15 minute to induce development of color. The reaction was terminated by the addition of stop solution and the resulting dye was measured in a spectrophotometer (Model 680 Micro plate Reader, Bio-Rad Laboratories Inc., Japan) at a wave length of 450 nm against the substrate blank. The IgE concentration of the samples was read from a standard curve. Mean values of two separate determinations from each sample was used as serum IgE level of a particular study subject.

### Statistical analysis

Data were analyzed using SPSS version 20 statistical package (SPSS, Inc., Chicago, IL, USA). A one-sample Kolmogorov-Smirnov test was used to assess whether the data were normally distributed. To determine the significance of differences between groups, we used unpaired *t*-test or Mann- Witney *U*-test if the data were not normally distributed. Post hoc Tukey test was used to determine which pairs of means differ significantly. Statistical significance was assigned for *p* values less than 0.05.

The z score values for height-, weight- and BMI-for-age relative to the WHO 2007 reference were calculated using Epi Info and WHO Anthro Plus softwares [18,19]. The z score values relative to the USCDC 2000 reference were calculated by the SPSS files provided by the USCDC [20]. Overweight (> + 1SD BMI-for-age z score), obesity (> + 2SD BMI-for-age z score), thinness/wasting (< −2SD of BMI-for-age z score), underweight (< −2SD of weight-for-age z score) and stunting (< −2SD of height-for-age z score) were defined according to the WHO and USCDC references. Weight-for-age is inadequate indicator for monitoring child growth beyond pre-school years due to its inability to distinguish between relative height and body mass, therefore, BMI-for-age is recommended by the WHO and USCDC to assess thinness/wasting in school-aged children and adolescents [21,22].

## Results

Four hundred five school children (218 boys and 187 girls) with mean age of 12.09 ± 2.54 were enrolled in this study. Anthropometric characteristics of the participating children based on absence or presence of intestinal infection are shown in Table 1. Intestinal infections were higher among younger school children with lower body weight and height compared to those without infection (p < 0.05).

**Table 1:** Comparison of anthropometric measurements of children with and without intestinal parasitic infection, Gondar, Northwest Ethiopia

	**Infected with**	**Not infected with intestinal**	**P value**
	**intestinal parasites**	**parasites**	
	**(n = 92, 22.7%)**	**(n = 313, 77.3%)**	
Boys (49/218)			
Age	11.51 ± 2.82	12.69 ± 2.55	0.006*
Height	136.33 ± 17.35	143.75 ± 15.60	0.005*
HAZ	−1.50 ± 1.59	−1.34 ± 1.08	0.4
Weight	31.87 ± 9.96	36.74 ± 10.25	0.004*
WAZ	−1.40 ± 1.43	−1.18 ± 0.86	0.2
BMI/age	−0.76 ± 1.77	−0.57 ± 1.06	0.3
Girls (43/187)			
Age	10.81 ± 2.22	12.08 ± 2.31	0.002*
Height	135.17 ± 13.45	140.08 ± 13.12	0.034*
HAZ	−1.23 ± 1.64	−1.40 ± 1.30	0.478
Weight	31.54 ± 8.16	36.21 ± 9.62	0.004*
WAZ	−0.90 ± 0.84	−0.89 ± 1.05	0.934
BMI/age	−0.55 ± 2.01	−0.19 ± 1.17	0.143

* Significantly different between infected and non infected (*P* < 0.05).

Results of stool examinations for intestinal parasites (Table 2) demonstrated the presence of a parasite in 22.7% of children surveyed, with a higher prevalence of intestinal parasites in children less than 13 years of age (85.9%) compared with those 13 years of age and above (14.1%). From the total parasitic infection, the most common intestinal parasite identified was *Ascaris lumbricoides* (48.1%), followed by *Hymenolepis nana* (28.3%), Hook worm (9.4%) and *Trichuris trichiura* (6.6%) in less than 13 years of age group. Mixed infection with more than one parasite was observed in 10 (2.6%) school children.

**Table 2:** Distribution of intestinal parasites in school children, Gondar, Northwest Ethiopia

**Organism**	**Number**	**Percentage**
*Ascaris lumbricoides*	51	48.1
*Hymenolepis nana*	30	28.3
Hook worm	10	9.4
*Trichuris trichiura*	7	6.6
*Enterobius vermicularis*	3	2.8
*Strongloids stecolaris*	2	1.9
*Giardia lamblia*	1	0.9
Entamoeba *spp.*	1	0.9
*Schistosoma mansonii*	1	0.9

Table 3 shows the prevalence of allergy stratified by sex, age, anthropometric values, parasite infection. In boys, the prevalence of allergy was higher at younger age and with low height-for-age z score compared to boys with higher score (p < 0.05). However, in girls, allergy were higher in older ages compared to younger ages (p < 0.05). 

**Table 3:** Characteristics of the participating children based on allergy, Gondar, Northwest Ethiopia

	**Without allergy**	**With allergy**	***P-value***
	**(n = 387)**	**(n = 18)**	
Boys			
Age	12.44 ± 2.62	12.25 ± 3.33	0.056*
Height	141.76 ± 16.24	147.59 ± 16.44	0.229
Weight	35.54 ± 10.25	37.53 ± 12.46	0.519
HAZ	−1.43 ± 1.21	−0.52 ± 0.96	0.011*
WAZ	−1.26 ± 1.03	−0.85 ± 0.82	0.176
BMIZ	−0.61 ± 1.27	−0.88 ± 0.87	0.460
Parasitic infection	0.21 ± 0.41	0.42 ± 0.51	0.102
Total IgE (IU/ml)	1809.59 ± 3037.425	1257.00 ± 1212.94	0.690
Girls			
Age	11.72 ± 2.25	14.00 ± 4.00	0.019*
Height	138.83 ± 13.28	142.67 ± 15.11	0.490
Weight	34.96 ± 9.15	40.58 ± 17.37	0.154
HAZ	−1.35 ± 1.40	−1.63 ± 0.58	0.637
WAZ	−0.89 ± 1.00	−1.06 ± 1.21	0.676
BMIZ	−0.27 ± 1.43	−0.32 ± 1.01	0.941
Parasitic infection	0.23 ± 0.42	0.33 ± 0.52	0.543
Total IgE (IU/ml)	2000.98 ± 3127.71	1267.67 ± 1793.02	0.692

* Significantly different between infected and non infected (*P* < 0.05).

In a subset of 100 participants, 80% without parasite and 20% with parasite infection, had median total serum IgE level 344 IU/ml (IQR 117–2076) and 610 IU/ml (IQR 143–1833), respectively (Z = −0.198, P > 0.8). The prevalence of self reported allergy among the subset was 8%. The Median IgE concentration in subjects without allergy 335 IU/ml (IQR 117–2076) and with history of allergy 610 IU/ml (IQR 394–1836) (Z = −0.813, P > 0.4) was not associated either with the presence of parasitic infection or history of allergy.

The prevalence of severe stunting, under weight and thinness/wasting (Z-score < −3) was found to be higher in girls 19 (10.2), 7 (3.7) and 7 (3.7) than boys 16 (7.3), 6 (2.8) and 6 (2.8), respectively. On the other hand, the percent values of moderate underweight and thinness/wasting (−3 to −2) was found to be higher in boys 30(13.8) and 17(7.8) than in girls 18(9.6) and 6(3.2), respectively (Table 4). The prevalence of moderate stunting was relatively similar in both sexes (16.5% boys and 16.6% girls). Overall, 102 (25.2%), 61 (15.1%) and 36 (8.9%) of the school children in Gondar were stunted, underweight and wasted, respectively, according to the reference criteria (z-score below –2SD) recommended by WHO. Severe (below –3SD z-score) stunting, underweight and wasting were found in 35 (8.6%), 13 (3.2%) and 13 (3.2%) of children, respectively.

**Table 4:** Distribution of school children according to z-score

	**Boys**			**Girls**		
**Z score**	**Height-for-age (%)**	**Weight-for-age (%)**	**BMI-for age (%)**	**Height-for-age (%)**	**Weight-for-age (%)**	**BMI-for age (%)**
<−3	16 (7.3)	6 (2.8)	6 (2.8)	19 (10.2)	7 (3.7)	7 (3.7)
−3 to −2	36 (16.5)	30 (13.8)	17 (7.8)	31 (16.6)	18 (9.6)	6 (3.2)
−2 to −1	85 (39.0)	94 (43.1)	54 (24.8)	59 (31.6)	48 (25.7)	25 (13.4)
−1 to 0	63 (28.9)	71 (32.6)	65 (29.8)	61 (32.6)	90 (48.1)	57 (30.5)
>0	18 (8.3)	17 (7.8)	76 (34.9)	17 (9.1)	24 (12.8)	92 (49.2)
Total	218 (100)	218 (100)	218 (100)	187 (100)	187 (100)	187 (100)

The status of malnutrition according to degrees of malnutrition is shown in Table 5. Cut off point of –2SD of the median for height-for-age z score, weight-for-age z score and BMI-for-age z score was calculated for determination of malnutrition. Although not statistically significant, higher number of stunting, under weigh and thinness/wasting were observed regardless of intestinal parasitic infections.

**Table 5:** The status of malnutrition in intestinal parasitic infected and non-infected children according to the cutoff point of -2SD Scores

		**Infected with intestinal parasites (92)**	**Not- infected (313)**
HAZ	Z-score ≤ −2SD	22 (23.9)	80 (25.6)
	(stunting)		
	Z-score > 2SD	70 (76.1)	233 (74.4)
WAZ	Z-score ≤ −2SD	15 (16.3)	46 (14.7)
	(under weight)		
	Z-score > 2SD	77 (83.7)	267 (85.3)
BMI/age	Z-score ≤ 2SD	10 (10.9)	26 (8.3)
	(thinness/wasting)		
	Z-score > 2SD	82 (89.1)	287 (91.7)

## Discussion

It has been estimated that more than 230 million (43%) of all preschool children in the developing world are stunted in their growth because of malnutrition caused by lack of food and by disease including parasitic infection [23]. In this study, despite the occurrence of a very high prevalence rate of intestinal parasitic infection among the school children, younger children with lower body weight and lower height were more infected with intestinal parasites than children with higher anthropometric parameters (*P* < 0.05). Reports have indicated that because parasitic infections such as soil transmitted helminthic infections cause anorexia and poor absorption of nutrients and promote the deviation of nutrients to the organism’s defense mechanisms; thereby contributing to the onset or exacerbation of weight and height deficits, as well as to specific nutritional deficiencies [24]. A number of earlier studies have shown that in the acute phase, these helminthic infections induce an immune response and the production of cytokines [25,26], which can directly affect the process of bone formation and remodeling required for the growth of long bones [27].

Intestinal parasites are highly prevalent among schoolchildren (55.6%-72.9%) in Northwest Ethiopia [28,29]. The present study however showed a relatively low prevalence of parasitosis (22.7%) comparable to the report from the study conducted in Babile town, eastern Ethiopia [30]. The difference could be due to variability in the prevalence of these infections, low sensitivity of the diagnostic method, the use of single stool sample and environmental contamination could partly explain the observed difference.

Although the prevalence rates of individual parasites vary considerably with altitude in different parts of the country, *A. lumbricoides*, *H. nana* and Hook worm were found to be the most prevalent parasites in this study. This finding was in agreement with reports of previous studies conducted in Ethiopia [30-32]; however, the prevalence of *H. nana* was higher than that of the study in Mexico and Pakistan [33,34]. The high rate of *H. nana* observed in this study indicates that hygienic practices of the schoolchildren in the study area are poor and that it is an important factor for auto-infection and transmission to others [35]. Unlike geo-helminths, *H. nana* does not require the external environment for maturation of eggs, which means the lifecycle of this parasite cannot be affected. The high prevalence might therefore be due to its high transmission.

Several studies have documented a positive association between BMI and allergy. Positive association between overweight and asthma was reported in black and Hispanic children [36]. In addition, in Taiwanese girls, a positive association was reported between the highest BMI quartile and risk for asthma symptoms, atopy and rhinitis [37]. Another study reported that a baseline BMI greater than 30 kg/m^2^ was a significant predictor of asthma incidence in women [38]. In the present study we evaluated several categorical (weight-for-age, height-for-age, BMI-for-age) and continuous (weight, height) anthropometric values, but only low height-for-age were related with allergy in boys. The present findings seem to be consistent with other research which found children with food allergies had weight-for-age and height-for-age Z scores lower than controls (0.1 versus 0.6 and 0.2 versus 0.8, respectively) [39].

Moreover, boys with allergy were younger than boys without allergy while girls with allergy were relatively older than girls without allergy. It is difficult to explain this result; hence, further research is needed to identify factors that contribute to such differences of this condition and to assess whether a similar pattern is also observed elsewhere.

In the present study, we evaluated total IgE responses, which are important components of host defense mechanisms against helminthic parasites in children and with differing degrees of malnutrition. In this study, high level of total IgE was observed; however, IgE concentration was not associated either with the presence of parasitic infection or history of allergy. The results are consistent with previous studies in Israeli-Ethiopian children and Vietnamese school children which found no significant difference in mean serum IgE between the stool parasite-positive group and the parasite-negative school children [40,41]. This also accords with our earlier observations, which showed that elevated IgE levels in diarrheic patients irrespective of HIV and/or intestinal parasitic infection [42].

A possible explanation for the high level of IgE observed irrespective of parasitic infection and history of allergy might be as is well established, malnutrition affects the immune response and increases the susceptibility to parasitic infection. On this regard, studies suggested that malnutrition and infectious agents that are frequent in malnourished children potentiates the polyclonal stimulation of IgE synthesis induced by helminths. As specific IgE antibody has been implicated in the resistance to helminthic infection, and the polyclonal stimulus diminishes this response, these factors may increase the susceptibility of malnourished children to such parasites [43,44]. Another possible contributing factor is a race-associated genetic mechanism; it was previously shown that IgE levels are higher in blacks than in Caucasians [45]. The effect of anti-helmintic treatment on the total serum IgE and specific IgE antibody response need to be elucidated in children with different nutritional status.

Undernutrition continues to be a cause of ill health and premature mortality among children in developing countries including Ethiopia [46]. This problem is not only associated with serious long-term consequences for the child but also adversely related to the economic development of a nation [47]. In the present study, the overall prevalence of underweight, stunting and thinness/wasting was 15.1%, 25.2% and 8.9% respectively. These prevalence rates of malnutrition indicated that the school children of this study area were in a better condition compared to malnutrition reported by a number of other studies [29,48-52]*.* Although the prevalence rates of malnutrition computed from the anthropometric measurements of the study children were not very severe compared to the national figure in 2005 which showed that underweight, wasted and stunted were 35.7, 9.7 and 51.3%, respectively [48], the prevalence of malnutrition remain substantial, demanding the attention of the responsible bodies.

The relationship of malnutrition and intestinal parasitic infection has been well established [9]. Different reports showed a close association between intestinal parasitism and malnutrition [53-55] which was not the case with this study*.* A possible explanation for this might be that both malnutrition and parasitic infections exist to a level of public health significance in the area, probably interacting synergistically and with other socio-economic and dietary factors [56].

### Limitations

This study has some limitations. First, the study was carried out in only urban schools of Northwest Ethiopia, and thus further investigation in suburban and rural areas is still needed. Second, the definition of allergy, which relies on self-reported physician diagnosed allergy. Third, there was no detailed information on socioeconomic status and non-availability of data on dietary intake. However, to our knowledge this article is the first of its type to look the relationship between allergy and nutritional status and, intestinal infection among school children in Ethiopia.

## Conclusion

The study provided baseline data about nutritional status, helminthic infections and prevalence of allergy in schoolchildren. Although prevalence of malnutrition was decreasing in the area [29,48], the prevalence of both malnutrition and intestinal parasitism was not negligible in this population. In addition, although helminthic infections can modulate the host inflammatory response directed against the parasite, we could not detect a significant association between the prevalence of allergy in this population and their nutritional status, and parasite infection. Further research prospective observational and intervention studies are required to address the question of causality between nutritional factors, parasites, and allergy.

## Abbreviations

ELISA: Enzyme linked immunosorbent assay; WHO: World health organization; BMI: Body mass index; HAZ: Height-for-age Z-score; WAZ: Weight-for-age Z-score; SPSS: Statistical package for social science; SD: Standard deviation; IQR: Inter quartile range.

## Competing interests

The authors declare that they have no competing interests.

## Authors’ contributions

AK: conception of the research idea, study design, data collection and analysis, interpret the data and reviewed the manuscript; BA, JA, BM, BF, GY, YB: study design, data collection, entry, analysis and drafting the manuscript; SG, DW, KT, EA, ME: data collection, part of laboratory work, data analysis and reviewed the manuscript; DT, AM, FO: helped in the statistical analysis, interpretation and reviewed the manuscript. All authors have read and approved of the final version of the manuscript.

## Pre-publication history

The pre-publication history for this paper can be accessed here:

http://www.biomedcentral.com/1471-2431/13/7/prepub
